# RNA interference gene therapy in dominant retinitis pigmentosa and cone-rod dystrophy mouse models caused by GCAP1 mutations

**DOI:** 10.3389/fnmol.2014.00025

**Published:** 2014-04-07

**Authors:** Li Jiang, Jeanne M. Frederick, Wolfgang Baehr

**Affiliations:** ^1^Department of Ophthalmology and Visual Sciences, John A. Moran Eye Center, University of Utah Health Science CenterSalt Lake City, UT, USA; ^2^Department of Biology, University of UtahSalt Lake City, UT, USA; ^3^Department of Neurobiology and Anatomy, University of Utah Health Science CenterSalt Lake City, UT, USA

**Keywords:** photoreceptor guanylate cyclase, guanylate cyclase-activating protein 1, short-hairpin RNA, RNA interference, self-complementary adeno-associated virus, cone-rod dystrophy, *retinitis pigmentosa*

## Abstract

RNA interference (RNAi) knockdown is an efficacious therapeutic strategy for silencing genes causative for dominant retinal dystrophies. To test this, we used self-complementary (sc) AAV2/8 vector to develop an RNAi-based therapy in two dominant retinal degeneration mouse models. The allele-specific model expresses transgenic bovine GCAP1(Y99C) establishing a rapid RP-like phenotype, whereas the nonallele-specific model expresses mouse GCAP1(L151F) producing a slowly progressing cone-rod dystrophy (CORD). The late onset GCAP1(L151F)-CORD mimics the dystrophy observed in human GCAP1-CORD patients. Subretinal injection of scAAV2/8 carrying shRNA expression cassettes specific for bovine or mouse guanylate cyclase-activating protein 1 (GCAP1) showed strong expression at 1 week post-injection. In both allele-specific [GCAP1(Y99C)-RP] and nonallele-specific [GCAP1(L151F)-CORD] models of dominant retinal dystrophy, RNAi-mediated gene silencing enhanced photoreceptor survival, delayed onset of degeneration and improved visual function. Such results provide a “proof of concept” toward effective RNAi-based gene therapy mediated by scAAV2/8 for dominant retinal disease based on GCAP1 mutation. Further, nonallele-specific RNAi knockdown of GCAP1 may prove generally applicable toward the rescue of any human GCAP1-based dominant cone-rod dystrophy.

## INTRODUCTION

Cone-rod dystrophies constitute a rare (1/40,000 prevalence) and heterogeneous class of hereditary retinal disease (Hamel, 2007). Symptoms of cone-rod dystrophy (CORD) may include photoaversion, attenuation of central visual acuity, achromatopsia, and eventually, extinction of peripheral vision. To date, ten genes are associated with dominant CORD: *PROM1* (Prominin-1)*, PRPH2* (Peripherin/rds)*, GUCA1A* (GCAP1)*, RIMS1* (Regulating Synaptic Membrane Exocytosis)*, GUCY2D* (Guanylate Cyclase 1)*, AIPL1* (Arylhydrocarbon-Interacting receptor Protein-Like 1)*, PITPNM3* (Phosphatidyl Inositol Transfer Membrane-associated family member 3)*, UNC119* (Uncoordinated 119 or HRG4)*, CRX* (Cone-Rod otX-like photoreceptor homeobox transcription factor), *and SEMA4A* (Semaphorin 4A; RETNET at https://sph.uth.edu/retnet/disease.htm). *GUCA1A*, encoding guanylate cyclase-activating protein 1 (GCAP1), is one of the most fully-characterized dominant CORD genes (Baehr and Palczewski, 2009) and involves about one dozen families with >100 affected members harboring various *GUCA1A* mutations (Jiang et al., 2011).

**FIGURE 1:**
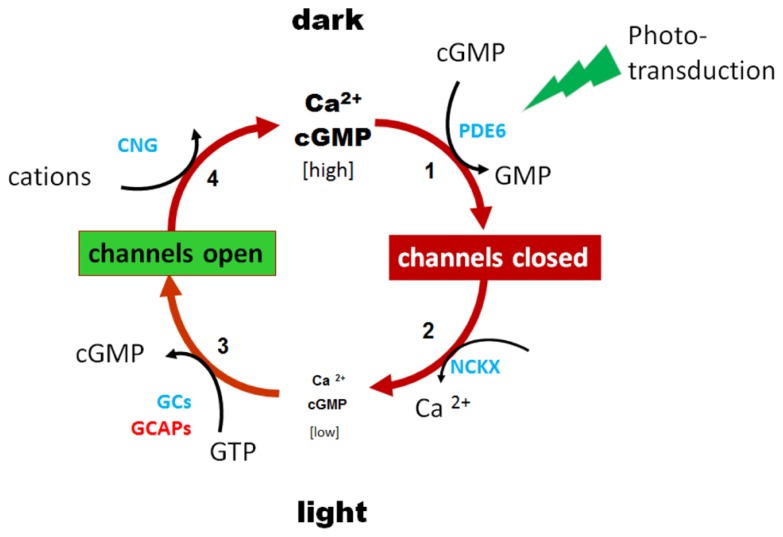
**Phototransduction feedback loop regulates levels of cGMP and Ca^**2**^^+^.** Dark levels of cGMP and Ca^2^^+^ are high in rod and cone photoreceptors. Light activation of rhodopsin initiates rod phototransduction, activated PDE6 rapidly hydrolyzes cytoplasmic cGMP, and cGMP-gated cation channels close. Continued extrusion of Ca^2^^+^ by the light-insensitive Na^+^-K^+^/Ca^2^^+^ exchanger (NCKX) lowers free Ca^2^^+^ which activates guanylate cyclase-activating proteins (GCAPs) and guanylate cyclases (GCs). Restoration of cGMP dark levels re-opens cation channels and Ca^2^^+^ levels equilibrate to dark levels.

Guanylate cyclase-activating protein 1 plays a key role in inhibiting photoreceptor guanylate cyclase activity at high free Ca^2^^+^, and accelerating guanylate cyclase activity in low free Ca^2^^+^. Hydrolysis of cyclic guanosine monophosphate (cGMP) by the phototransduction cascade closes cGMP-gated channels, reducing influx of Ca^2^^+^ ions (**Figure 1**). Continuous extrusion of Ca^2^^+^ by the light-insensitive NCKX exchanger lowers cytoplasmic Ca^2^^+^, thereby activating GCAP1, and GC and re-establishing dark cGMP levels. In rods, cGMP levels are regulated by two guanylate cyclases (GC1 and GC2; Goraczniak et al., 1994, 1997; Lowe et al., 1995; Duda et al., 1996) and two GCAPs (GCAP1 and GCAP2; Dizhoor et al., 1994, 1995; Palczewski et al., 1994; Gorczyca et al., 1995), while cone phototransduction relies on GC1 and GCAP1 exclusively. The two GCAPs overlap partially in regulating the GCs of rods (Makino et al., 2008; Dizhoor et al., 2010; Peshenko et al., 2011), but with differential signaling modes (Duda et al., 2005, 2012). Both GCAPs contribute to rod recovery after photolysis (Mendez et al., 2001; Howes et al., 2002; Makino et al., 2008, 2012). Germline deletion of both GCAPs renders GC activity in rods and cones Ca^2^^+^-insensitive (Mendez et al., 2001). Transgenic GCAP1 could restore normal rod and cone response recovery (Howes et al., 2002; Pennesi et al., 2003).

**FIGURE 2:**
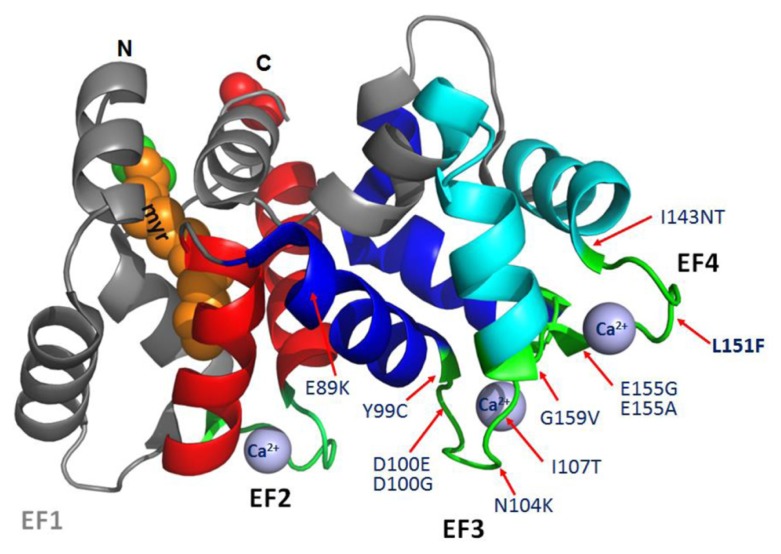
**Structure of myristoylated GCAP1 (PDB 2R2I; Baehr and Palczewski, 2009)**. N, N-terminal. C, C-terminal. EF hand helix-loop-helix structures are shown: EF1 (inactive and gray), EF2 (red), EF3 (dark blue), and EF4 (turquoise). Ca^2^^+^-binding loops (green) and approximate positions of relevant missense mutations associated with adCORD (red arrows) are indicated. The N-terminal myristoyl group (orange–brown) is buried and nearly invisible.

GCAPs feature four EF hand motifs, of which three (EF2-4) are high-affinity Ca^2^^+^ binding sites and one (EF1) is inactive (Palczewski et al., 2004; Baehr and Palczewski, 2009). Six missense mutations in GCAP1 associated with autosomal dominant CORD3 are found in EF3 (E89K, Y99C, D100E, D100G, N104K, I107T; Dizhoor et al., 1998; Payne et al., 1998; Sokal et al., 1998; Jiang et al., 2008; Kitiratschky et al., 2009; Kamenarova et al., 2013; Nong et al., 2014). In EF4, only four missense mutations have been identified (I143NT, L151F, E155G, E155A, G159V; Wilkie et al., 2001; Nishiguchi et al., 2004; Jiang et al., 2005; Sokal et al., 2005; Huang et al., 2013; **Figure 2**). These dominant GCAP1 mutations alter Ca^2^^+^-association, decrease Ca^2^^+^ sensitivity, and produce constitutive activity of photoreceptor GC1 at normal “dark” Ca^2^^+^ levels. Persistent stimulation of GC1 in the dark increases cGMP to toxic levels. (Dizhoor et al., 1998; Sokal et al., 1998; Woodruff et al., 2007). Elevated levels of cGMP open more CNG channels, elevate free Ca^2^^+^ in outer segments, and trigger cell death by unknown mechanisms. Elevated Ca^2^^+^ levels have been suspected to trigger cell death, however a massive accumulation of cGMP in *Cnga3^-^^/^^-^*;*Nrl^-^^/^^-^* mice lacking cation channels and elevated Ca^2^^+^ also correlated with photoreceptor apoptotic death. This result excludes Ca^2^^+^ as a death trigger and supports a role of cGMP accumulation as the major contributor to cone death and a role cGMP-dependent protein kinase G (PKG) regulation in cell death (Xu et al., 2013).

In a four-generation British family, CORD was mapped to chromosome 6p21.1 (Payne et al., 1998). The disease-causing mutation was identified as Y99C, a change that was absent in over 200 unrelated controls. The same mutation was later identified independently in two ancestrally related families (Downes et al., 2001). The Y99C mutation in GCAP1 has also been reported to cause isolated macular dysfunction (Michaelides et al., 2005). Thus far, families presenting with cone and CORD have been independently linked to a GCAP1(L151F) mutation. In the first family (Sokal et al., 2005), hemeralopia, dyschromatopsia and reduced visual acuity became evident by the second-to-third decades of life with non-recordable photopic ERG responses. In the second pedigree spanning five-generations (Jiang et al., 2005), 11 of 24 individuals displayed photoaversion, color vision defects, and central acuity loss with onset of legal blindness during the second-to-third decades of life. The GCAP1(L151F) gene product, known to disrupt Ca^2^^+^ coordination at EF-hand four, and alter Ca^2^^+^ sensitivity (Sokal et al., 2005), represents a conservative substitution.

## GCAP1 MUTANT MOUSE MODELS

**FIGURE 3:**
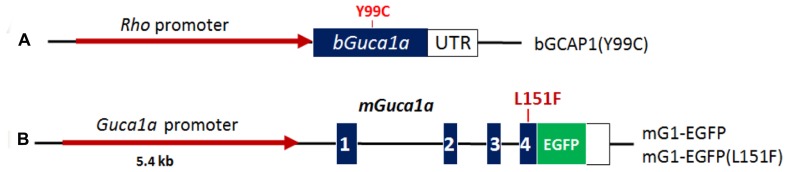
**Schematic representation of GCAP1 transgenes used to generate RP and CORD mouse models. (A)**, bovine GCAP1(Y99C) transgene. Bovine *Guca1a* cDNA (blue) is expressed under the control of the Rho promoter (red). Approximate position of the Y99C mutation in EF3 is indicated (Jiang et al., 2011). **(B)**, mouse GCAP1 transgene under the control of its native promoter (red). Exons (blue) are numbered; the L151F mutation is located in exon 4 and EGFP is fused in-frame to the GCAP1 C-terminus [see **Figure 2** (Jiang et al., 2013)].

To study GCAP1-associated retinal degeneration disease, several GCAP1 mouse models have been generated, expressing different GCAP1 mutants. The first generated GCAP1 transgenic mice expressing bovine GCAP1(Y99C) mutant gene displayed severe *retinitis pigmentosa*-like phenotypes as the mutant transgene was specifically expressed in rod photoreceptors under control of a rhodopsin promoter (Olshevskaya et al., 2004). A GCAP1(E155G) knock-in mouse could mimick human patients and presented late-onset and slowly progressive cone-rod photoreceptor degeneration (Buch et al., 2011). Expressing GCAP1(E155G) in mouse rods also caused severe early-onset rod-cone degeneration (Woodruff et al., 2007). Our laboratory generated three transgenic mouse lines expressing wild-type GCAP1-EGFP, as well as mutant GCAP1(L151F), and GCAP1(L151F)-EGFP (Jiang et al., 2013; **Figure 3**). The three transgenes were modified from mouse genomic *Guca1a* gene fragment, containing its native regulatory elements. They are expressed at a level comparable with endogenous GCAP1 in the transgenic mice, and the mutant transgenic mice develop retina pathology slowly and recapitulate features of human CORD (Jiang et al., 2013).

Our aim was to develop first a self-complementary AAV2/8 (scAAV2/8) knockdown virus that expresses an allele-specific short hairpin RNA (shRNA) targeting the mutant *Guca1a* gene in the GCAP1(Y99C) transgenic mice. Further, we intended to target both mutant and native *Guca1a* in GCAP1(L151F)-EGFP transgenic mice (nonallele-specific shRNA targeting). In the first set, we used two transgenic mouse lines expressing bGCAP1(Y99C) cDNA under the control of the rhodopsin promoter (**Figure 3**). One line expressed GCAP1 at near normal levels and the second line overexpressed GCAP1 (3–4X; Olshevskaya et al., 2004). These lines are models for dominant *retinitis* *pigmentosa* representing moderate (line L52H) or severe (line L53) retinal degeneration phenotypes (Olshevskaya et al., 2004). An allele-specific knockdown bGCAP1(Y99C) is expected to significantly slow down the dystrophy and even delay onset of degeneration.

The second set of transgenic mice contained the L151F mutation introduced in exon 4 of the *Guca1a* gene. We employed the entire *Guca1a* gene including promoter, all exons and introns, and the 3′-UTR containing the polyadenylation signals. We established two mutant GCAP1 mouse lines, GCAP1(L151F), and GCAP1(L151F)-GFP (**Figure 3**). A third line expressing a GCAP1-EGFP fusion protein (no L151 mutation) served as a WT control (**Figure 3**). The EGFP tag allowed detection of transgene expression by live fluorescence microscopy, and distinction of transgenic GCAP1-EGFP (50 kDa) from native GCAP1 (23 kDa). We intended to knockdown both WT and mutant GCAP1s as removal of photoreceptor GCAP1 does not affect retina development or morphology, and GCAP1/2 double knockout mice do not exhibit retina degeneration.

## *IN VITRO* SCREENING FOR POTENT shRNA SEQUENCES

**FIGURE 4:**
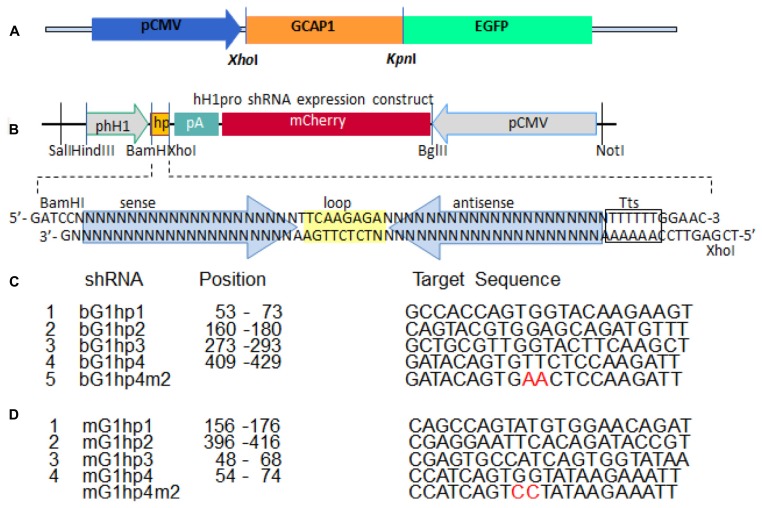
***In vitro* screening strategy to identify a potent shRNA knockdown sequence. (A)**, a construct expressing the GCAP1-EGFP fusion protein under the control of the CMV promoter. **(B)**, the shRNA expression construct. shRNA (hp yellow box) is under the control of the H1 promoter, and the reporter gene mCherry is under the control of the CMV promoter. Hp consists of a sense-loop-antisense construct (the hairpin). Tts, transcription terminator signal. **(C)**, four candidate shRNAs for suppression of bovine GCAP1, and a 2-nucleotide mismatch control. **(D)**, four candidate shRNAs for suppression of both wild-type and mutant mouse Guca1a mRNA, and a 2-nucleotide mismatch control. See also **Figure 1** (Jiang et al., 2011), and **Figure 6** (Jiang et al., 2013).

For successful knockdown via shRNA, it is essential to screen candidate knockdown sequences for specificity and efficacy. We developed an *in vitro* screening strategy consisting of a shRNA expression vector with reporter gene, to be co-expressed with a second vector expressing GCAP1-GFP as a prey (**Figures 4A,B**). This *in vitro* screening system permits the testing of a number of candidates and eventual identification of the most powerful shRNA. The shRNA vector, hH1pro, contained an shRNA expression cassette driven by a human H1 pol-III promoter, and a CMV-driven mCherry reporter gene arranged in a tail-to-tail array (**Figure 4B**). For allele-specific targeting we generated four shRNA constructs, bovine (b) G1hp1-4, expressing four candidate anti-bovine GCAP1 shRNAs specifically targeting b*Guca1a* mRNA (**Figure 4C**). Similarly, we generated four anti-mouse GCAP1 shRNA expression constructs, mG1hp1-4, for nonallele-specific targeting of both transgenic and endogenous mouse GCAP1 mRNAs. (**Figure 4D**).

**FIGURE 5:**
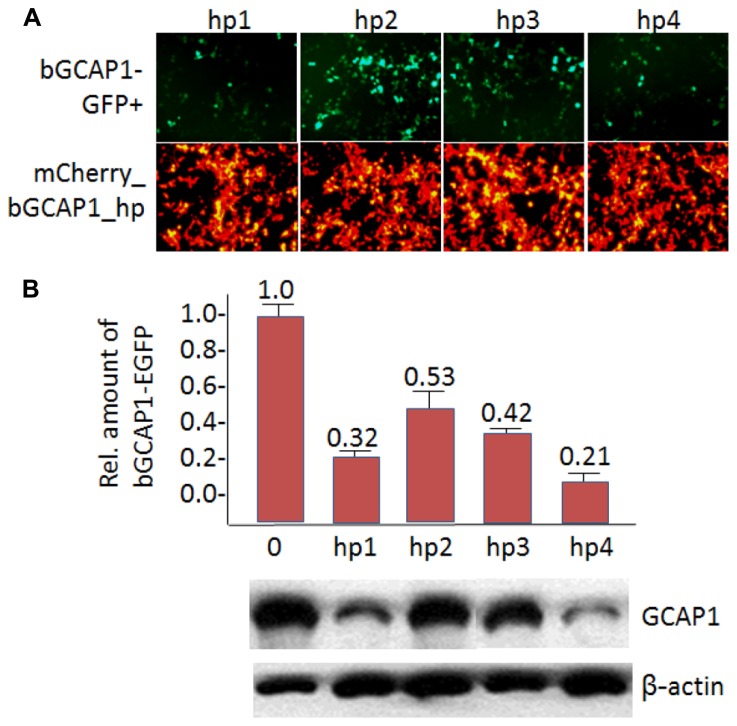
***In vitro* knockdown of bGCAP1-EGFP. (A)**, *In vitro* transfection of HEK cells expressing bGCAP1-GFP (top) with bG1hp1-4 (bottom). Top panels show that hp1 and hp4 are highly effective. Bottom panels demonstrate fairly uniform transfection of four shRNA expression plasmids. **(B)**, Representative semi-quantitative immunoblot of GCAP1 knockdown. Bar graph (top, *n* = 3) identifies hp4 as the most potent shRNA. See also **Figure 2** (Jiang et al., 2011), and **Figure 7** (Jiang et al., 2013).

In HEK293 cell culture, we tested the knockdown efficiencies of four putative bGCAP1 shRNA by cotransfection of each expression construct (bG1hp1-4) with the target bGCAP1-EGFP (**Figure 5A**). The expression levels of bGCAP1-EGFP in the transfected cells were detected by live cell fluorescence microscopy and immunoblotting assay 48–50 h after cotransfection. Under a similar expression level indicating by the mCherry reporter (**Figure 5A**, lower panel), four bGCAP1 shRNA candidates suppressed GCAP1-GFP expression at different efficiencies (**Figure 5A**, upper panel). Among them, bG1hp4 was shown the highest knockdown efficiency, presented by the lowest GFP fluorescence in the cotransfected cell cultures, and 79% reduction of GCAP1-GFP compared to 68, 47, and 58% for other three shRNAs in semi-quantitative immunochemistry assay (**Figure 5B**). By using the same *in vitro* screening experiment, we demonstrated that among four anti-mouse GCAP1 shRNAs (mG1hp1-4), mG1hp4 is the most efficient, as it deceased mGCAP1-GFP expression by more than 70% in cell culture. Knockdown specificities of bG1hp4 and mG1hp4 were tested with their 2-nucleotide mismatch shRNA controls, bG1hp4m2 and mG1hp4m2. Compared to bG1hp4 and mG1hp4, the respective mismatch controls were unable to suppress expression of co-transfected bGCAP1-GFP and mGCAP1-GFP (**Figure 4**).

## GENERATION OF SELF-COMPLEMENTARY ADENO-ASSOCIATED VIRUS

**FIGURE 6:**
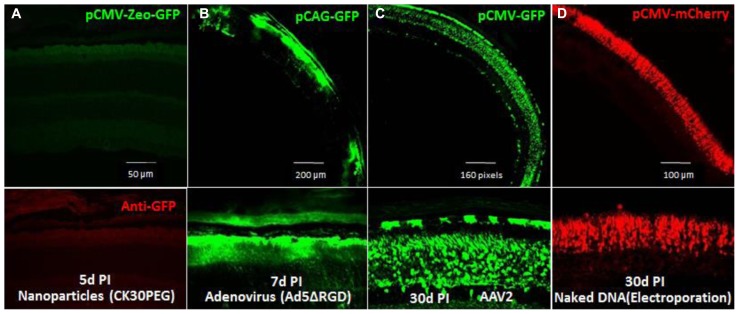
**Transduction efficiencies of four gene delivery methods for mouse photoreceptors tested by expression of fluorescent protein reporter. (A)**, Fluorescence microscopy of retina transverse-sections of mice injected subretinally with pCMV-Zeo-GFP, peptide/polymer nanoparticles. Below, retina section probed with anti-GFP antibody. **(B)**, Fluorescence microscopy of retina transfected with pCAG-GFP using recombinant adenovirus 5, Ad5ΔRGD. Below, section at higher magnification. **(C)**, pCMV-GFP-transfected retina using recombinant AAV vector, AAV2. Underneath, section at higher magnification. **(D)**, pCMV-mCherry-transfected retina using electroporation. Micrographs were recorded at various days post-injection (PI).

To efficiently express the shRNAs in mouse photoreceptors, we tested four currently used gene delivery vectors: a peptide/polymer nanoparticle (CK30PEG; Farjo et al., 2006), recombinant adenovirus (Ad5ΔRGD; Cashman et al., 2007; Sweigard et al., 2010), recombinant AAV2 (Wu et al., 2006), and electroporation with naked DNA. No GFP reporter gene expression was detected in the CK30PEG-transduced retina (**Figure 6A**), and the Ad5ΔRGD-transduced retina had much more robust GFP expression in RPE cells than in photoreceptors (**Figure 6B**). The AAV2-transduced retina showed the most robust GFP expression in photoreceptors (**Figure 6C**), suggesting AAV vector is a highly efficient gene vector for shRNA delivery to mouse photoreceptors. Although neonatal electroporation of subretinally injected plasmid DNA showed specific and efficient photoreceptor transfection (**Figure 6D**), this method is only useful for undifferentiated and mitotic photoreceptors and is therefore, not applicable for human gene therapy.

**FIGURE 7:**
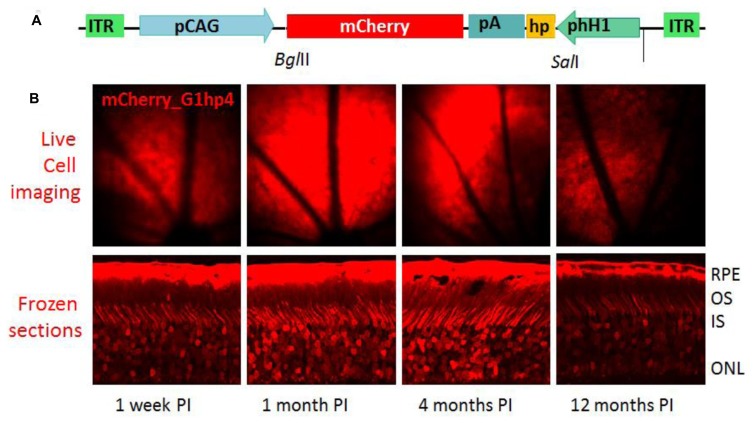
**Long-term expression of shRNA *in vivo*. (A)**, Schematic of scAAV2/8 shuttle vector for anti-bGCAP1 shRNA. ITR, inverted terminal repeats; hH1, human H1 promoter; hp, shRNA cassette; pCAG, chicken actin promoter driving mCherry. **(B)**, Upper panel, *in vivo* fluorescence fundus images of wild-type mice at 1 week and months (indicated) post-subretinal injection of scAAVhp4 viral particles. Lower panel, fluorescence microscopy of corresponding mouse retina transverse cryosections (Jiang et al., 2011). RPE, retinal pigmented epithelium; OS, outer segments; IS, inner segments; ONL, outer nuclear layer.

Among currently available AAV vectors, the scAAV vector, scAAV2/8, shows the most effective and stable transgene expression in mouse photoreceptors (Natkunarajah et al., 2008). We packaged the shRNA expression cassette with an mCherry reporter into the recombinant viral vector to generate two scAAV2/8 viral constructs expressing shRNAs targeting bovine and mouse GCAP1s, respectively, scAAVbG1hp4 and scAAVmG1hp4. By examining the mCherry reporter expressed in the scAAVbG1hp4-transduced mouse retinas, we demonstrated that scAAV2/8 vector could generate a long-term transgene expression lasting to 1 year (**Figure 7**).

## THERAPEUTIC EFFECTS OF KNOCKDOWN VIRUS

**FIGURE 8:**
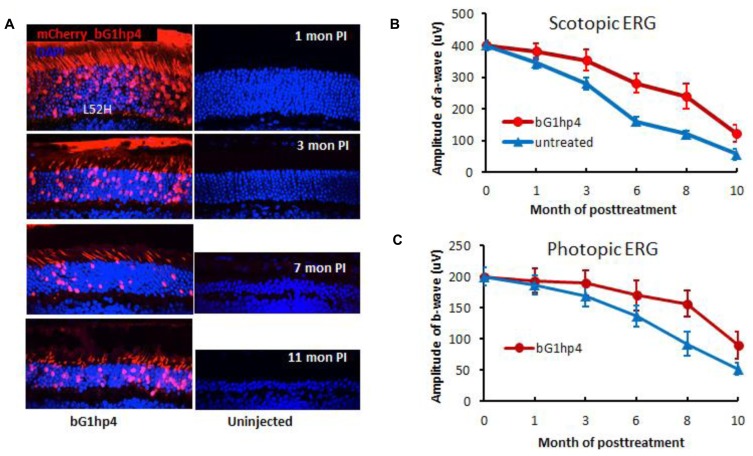
**Long-term therapeutic efficiency of allele-specific shRNA vector, scAAV2/8-bG1hp4. (A)**, Direct fluorescence microscopy of retinal cross-sections examines retinal morphology of the bG1hp4 treated and untreated L52H transgenic mice. Viral vectors were injected subretinally at mouse ages P21–P30. Both treated and untreated eyes were harvested at four representative times from 1–11 months post-injection. Red, mCherry expression demonstrating scAAV2/8 virus transduction. Blue, DAPI staining of nuclei. Note significant preservation of ONL thickness at 11 months post-treatment (~12 months of age) compared to non-treated controls. **(B,C)**. Scotopic and photopic ERG amplitudes recorded from bG1hp4-treated (red) and untreated (blue) L52H transgenic mice (Jiang et al., 2011). Subretinal injection of scAAV2/8-bG1hp4 in the transgenic mouse models delayed progression of both rod and cone dysfunction significantly.

To test allele-specific knockdown of GCAP1 *in vivo*, we injected the scAAVbG1hp4 virus into the mouse subretinal space using two different bGCAP1(Y99C) transgenic mouse lines, L53, and L52H. Expression of the mutant bGCAP1(Y99C), but not that of endogenous mouse GCAP1, was significantly suppressed by scAAV2/8-mediated bG1hp4 expression in the retinas of transgenic mice. In the severe and rapid retinal degeneration mouse line L53, bG1hp4 significantly delayed photoreceptor cell death, which was observed at 30 and 45 days post-injection. With the moderate retinal degeneration line L52H, we demonstrated a long-term therapeutic effect of scAAVbG1hp4 virus from 1 month up to 11 months post-injection, assayed by retinal morphology (**Figure 8A**) and function (**Figures 8B,C**).

**FIGURE 9:**
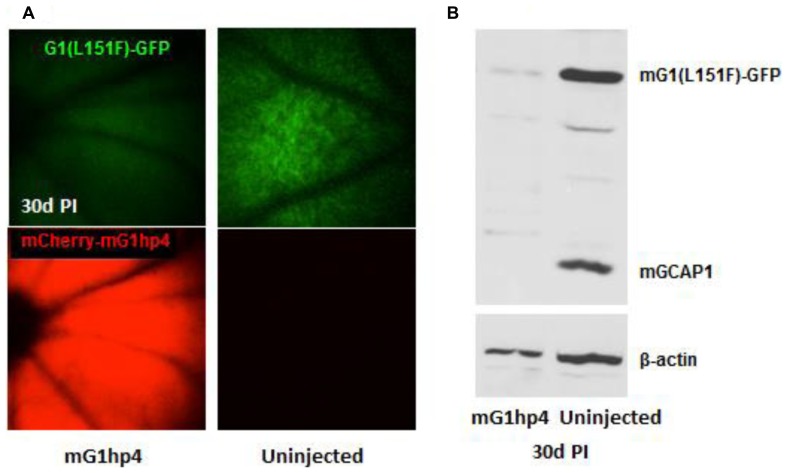
**Knockdown of mGCAP1 by nonallele-specific shRNA vector, scAAV2/8-mG1hp4. (A)**, Fluorescence fundus images of mG1(L151F)-GFP transgenic mice with subretinal injection of the virus vectors at P21–P30 (left) and non-injected control (right). GFP signal (top) represents the mG1(L151F)-GFP expression level in photoreceptors, and mCherry (bottom) signal indicates scAAV2/8 virus transduction. **(B)**, Immunoblot of the transgenic mG1(L151F)-GFP and endogenous GCAP1 protein levels in the injected and the non-injected retinas at 30 days PI. Transgenic mG1(L151F)-GFP (~50 kD) and endogenous GCAP1 (~25 kD) proteins were detected by UW101 antibody directed against GCAP1. -actin served as endogenous loading control. See also **Figure 9** (Jiang et al., 2013).

As deletion of GCAPs in mouse apparently has no detrimental morphological nor disease-causing defect, except for increased sensitivity and delayed recovery from the dark state (Mendez et al., 2001), we assumed that nonallele-specific shRNA knockdown of both wild-type and mutant GCAP1s may be a general therapeutic strategy to treat retinal degeneration in patients carrying GCAP1 mutations. We tested *in vivo* knockdown efficiency of scAAV2/8-mediated mG1hp4 in the mGCAP1(L151F)-GFP transgenic mice. Fluorescence fundus imaging indicated significant suppression of the mGCAP1(L151F)-GFP mutant transgene, but not in uninjected eyes (**Figure 9A**). Broad and robust mCherry reporter gene expression in scAAVmG1hp4-treated mouse retinas signaled even expression levels of the virus. Semi-quantitative analysis of GCAP1 levels in treated retinas revealed that mG1hp4 simultaneously knocked down *in vivo* expression of the mutant mGCAP1(L151F)-GFP transgene by 70% and endogenous wild-type GCAP1 by 90% (**Figure 9B**). Thus our experiment provides “proof of principle” that nonallele-specific RNA interference (RNAi) knockdown may be a strategy applicable to all GCAP1 mutations.

## CONCLUSION

The experimental goal was to test whether allele-specific or nonallele-specific knockdown of a dominant GCAP1 mutant is able to ameliorate photoreceptor dystrophy. We demonstrated the feasibility of shRNA knockdown first with an allele-specific approach in a *retinitis pigmentosa* mouse model expressing GCAP1(Y99C). A scAAV robustly expressed shRNAs in photoreceptors at 1 week post-injection and gene silencing activity persisted as long as 1 year without any apparent off-target interference. Delayed disease onset, significantly improved rod photoreceptor survival and increased visual function support that the methodology can be useful for human gene therapy.

For nonallele-specific knockdown, we generated a sophisticated set of transgenic mouse models expressing GCAP1-EGFP fusion proteins with and without L151F mutation. The L151F mutation, discovered in our lab, was shown to cause dystrophy in two unrelated families. Nonallele-specific shRNA knockdown of both wild-type and mutant GCAP1s may serve as a therapeutic strategy to rescue the dominant degeneration caused by any of the eleven known EF-hand GCAP1 mutations. An advantage of dominant GCAP1 mutations is that a nonalle-specific approach promises to be successful, while mutations in other CORD genes require gene replacement to rescue the disease. Successful knockdown by RNAi suppression of both wild-type and mutant GCAP1s may be a potent therapeutic strategy, applicable to all affected family members with CORD based on GCAP1 mutations in EF3 and EF4, as long as the shRNA guide strand is located external to the disease-causing mutations.

## Conflict of Interest Statement

The authors declare that the research was conducted in the absence of any commercial or financial relationships that could be construed as a potential conflict of interest.
